# Work characteristics and mental health among physicians in Bangladesh: highlighting gendered perspectives

**DOI:** 10.1192/bji.2025.10075

**Published:** 2026-11

**Authors:** Afsari Banu Alpona, Sadia Afrin Rupa, Randy Deny Kurniawan, Mengjiao Yang, Smarika Shrestha, Mingyu Cui, Mengxuan Wang, Yolanda De Fatima De Oliveira Graca, Hilda Meriyandah, Tokie Anme

**Affiliations:** 1 PhD student, Doctoral Program in Medical Sciences, Graduate School of Comprehensive Human Sciences, University of Tsukuba, Japan; 2 Study Physician, Research, Projahnmo Research Foundation, Manikganj, Bangladesh; 3 Master’s student, Master’s Program in Public Health, Graduate School of Comprehensive Human Sciences, University of Tsukuba, Japan; 4 Nurse, Cardiovascular Surgery, North Sichuan Medical College Hospital, Nanchong, China; 5 PhD student, Doctoral Program in Medical Sciences, Graduate School of Comprehensive Human Sciences, University of Tsukuba, Japan; 6 Postdoctoral Researcher, Food Hygiene and Nutrition, School of Public Health, Peking University, Beijing, China; 7 PhD student, Doctoral Program in Medical Sciences, Graduate School of Comprehensive Human Sciences, University of Tsukuba, Japan; 8 PhD student, Doctoral Program in Medical Sciences, Graduate School of Comprehensive Human Sciences, University of Tsukuba, Japan; 9 PhD student, Doctoral Program in Medical Sciences, Graduate School of Comprehensive Human Sciences, University of Tsukuba, Japan; 10 Professor, Faculty of Medicine, University of Tsukuba, Ibaraki, Japan.

**Keywords:** Working conditions, mental health, male, female, physicians

## Abstract

**Background:**

Work characteristics play a crucial role in the mental well-being of physicians. However, limited research in Bangladesh has explored the association between these characteristics and specific mental health outcomes such as depression, anxiety and stress among physicians, particularly in relation to gender differences.

**Aims:**

This study aimed to explore the link between various work characteristics and mental health outcomes among male and female physicians in Bangladesh.

**Method:**

We conducted a cross-sectional study among physicians working in various healthcare settings in Bangladesh. The data were collected online between November 2023 and January 2024 using a convenience sampling technique. Work characteristics, including job characteristics, social characteristics and organisational characteristics, were assessed using previously validated scales. Mental health, on the other hand, was measured using the Depression, Anxiety and Stress Scale (DASS-21). We performed logistic regression analyses adjusted for the covariates, and further stratified by gender, to explore potential differences in work characteristics and mental health outcomes between male and female physicians.

**Results:**

In our study, social characteristics were significantly inversely associated with depression (adjusted odds ratio 0.37 (0.20–0.71)), anxiety (adjusted odds ratio 0.53 (0.30–0.92)) and stress (adjusted odds ratio 0.45 (0.26–0.81)). Organisational characteristics showed a significant inverse association only with stress (adjusted odds ratio 0.42 (0.24–0.74)). Among male physicians, organisational characteristics were significantly inversely associated with depression (adjusted odds ratio 0.42 (0.19–0.90)), anxiety (adjusted odds ratio 0.44 (0.21–0.91)) and stress (adjusted odds ratio 0.42 (0.20–0.89)), while social characteristics were significantly inversely linked only to stress (adjusted odds ratio 0.43 (0.19–0.97)). By contrast, among female physicians, only social characteristics demonstrated a significant inverse association with depression (adjusted odds ratio 0.30 (0.12–0.78)).

**Conclusions:**

This study highlights the importance of social characteristics as a protective factor for psychological well-being in the healthcare context. Therefore, fostering a work culture that prioritises peer support and strong interpersonal relationships can be crucial in alleviating mental health challenges among physicians.

The World Health Organization reported in 2025 that more than 1 billion people worldwide suffer from a mental health issue, with anxiety and depression being the most common, across all ages and all regions.^1^ This global burden of mental health issues was exacerbated during the COVID-19 pandemic, with an estimated 53.2 million additional cases of major depressive disorder (MDD) worldwide, representing a 27.6% increase (25.1–30.3), and 76.2 million additional cases of anxiety disorders, marking a 25.6% rise (23.2–28.0). South Asia alone experienced a significant increase of 36.1% in MDD and 35.1% in anxiety disorders.^2^

In Bangladesh, mental disorders have long been neglected, under-prioritised, not seen as serious health issues and considered as stigma, taboo or negative career repercussions.^3–5^ During the COVID-19 pandemic, the reported prevalence of anxiety was 33.7%, of depressive symptoms was 57.9% and of stress was 59.7%.^6^ Following the COVID-19 outbreak, mental health issues among physicians were put under the spotlight, revealing a prevalence higher than that among the general population.^4^ Previous studies reported that the following determinants majorly contributed to depression, anxiety and stress among Bangladeshi physicians: being younger; being female; having a history of diagnosed mental health issues; having comorbidities; having low salaries and incentives; having inadequate managerial or organisational support; and fear related to the COVID-19 pandemic.^4,7,8^ While previous studies have acknowledged poor working conditions in the healthcare sector,^9^ the specific work-related factors contributing to depression, anxiety and stress among Bangladeshi physicians remain underexplored.

Work characteristics encompass career, social and organisational components that influence the well-being of employees and organisational objectives.^10^ Job characteristics relate to the various facets and components of a job that affect employee satisfaction and motivation, such as autonomy, feedback and task significance.^11^ While task-related aspects are addressed by job characteristics, the social aspect of work is equally important, particularly for occupations that depend on interpersonal interactions. Social characteristics refer to the attributes that concern the interpersonal and relational dynamics involved in performing work in the workplace.^10,12^ In addition to individual and relational dimensions, organisational attributes play a broader role in shaping the work experience via structural and institutional elements. Organisational characteristics refer to work context characteristics, which can be described as the managerial and demographic variables that subsequently and partially comprise its internal environment.^13^ The relationship between work characteristics and mental health is complex, involving not only organisational factors but also aspects of the job itself and social dynamics. Previous research underscores that strategic work design, including autonomy, task variety and workload optimisation, is essential for enhancing employees’ attitudes, behaviours and psychological well-being, which ultimately aids in achieving both individual and organisational goals.^10,12^ This dynamic can be explained using the job demand–resources (JD-R) model.^14^ According to this model, the interplay between job demands, such as physical and mental efforts, and job resources, such as autonomy and organisational support, determines employee well-being and performance.^14,15^ High job demands, coupled with low job control and insufficient rewards, can lead to increased anxiety and depression.^16^ While prior research has explored the relationships between work characteristics and job satisfaction, turnover intention and burnout among physicians,^10^ there have been limited numbers of studies investigating the association between these characteristics and depression, anxiety and stress, particularly concerning gender differences. Bangladeshi female physicians encounter further challenges in their workplace due to the predominant patriarchal society.^17^ Despite outnumbering male physicians,^5^ they experience 2.16 times more stress than their male colleagues.^7^ The societal environment in Bangladesh makes it challenging for female physicians to maintain a healthy balance between work and personal life.^18^ These issues are exacerbated by poor working conditions, inadequate resources, lack of organisational support, a limited workforce, a poor physical work environment, workplace violence, etc.^9,10^

This study aims to fill the gap by exploring the relationships between various work characteristics and depression, anxiety and stress among Bangladeshi physicians, with a particular focus on gender differences. Understanding these associations could facilitate the development of targeted prevention strategies and interventions to protect and prioritise the mental health of physicians, particularly within the context of low- and middle-income countries (LMICs).

## Method

### Study design, population and samples

This study utilised a cross-sectional, self-report survey conducted among physicians in various healthcare settings in Bangladesh. A convenience sampling method was employed, and participation was voluntary. The eligibility criteria were registered physicians residing and working in Bangladesh, possessing at least 1 year of work experience and having no diagnosis of psychiatric illness or use of psychotropic medications. A total of 388 physicians participated in the survey. Sample size was calculated using a single proportion formula for an infinite population. Assuming a 67.7% prevalence of anxiety among physicians in Bangladesh,^4^ with a 95% confidence interval and 5% margin of error, the required sample size was 336.

### Data collection

Because mental health is a sensitive topic in Bangladesh, we designed our survey to be online and anonymous to protect participants’ identity, encouraging honest and uninhibited responses. The survey, created using LimeSurvey (LimeSurvey GmbH, Hamburg, Germany; see https://www.limesurvey.org/), was disseminated through an online forum for Bangladeshi physicians, as well as for medical and dental students, called Platform. This is the largest social media forum for Bangladeshi physicians, managed by the medical community and dedicated to enhancing physicians’ quality of life through support, advocacy and community building.^7^ We followed the Checklist for Reporting Results of Internet E-Surveys (CHERRIES) guidelines.^19^ The survey was active for 3 months, from November 2023 to January 2024.

The consent form was presented at the beginning of the survey, requiring participants to agree to participate before proceeding. Selecting ‘I agree to participate’ directed them to the first page of the questionnaire, while selecting ‘I do not agree to participate’ ended the session. Our e-questionnaire consisted of three major sections: demographic characteristics, work characteristics and mental health-related questions (see Supplementary Material 1 available at https://doi.org/10.1192/bji.2025.10075).

**Fig. 1:**
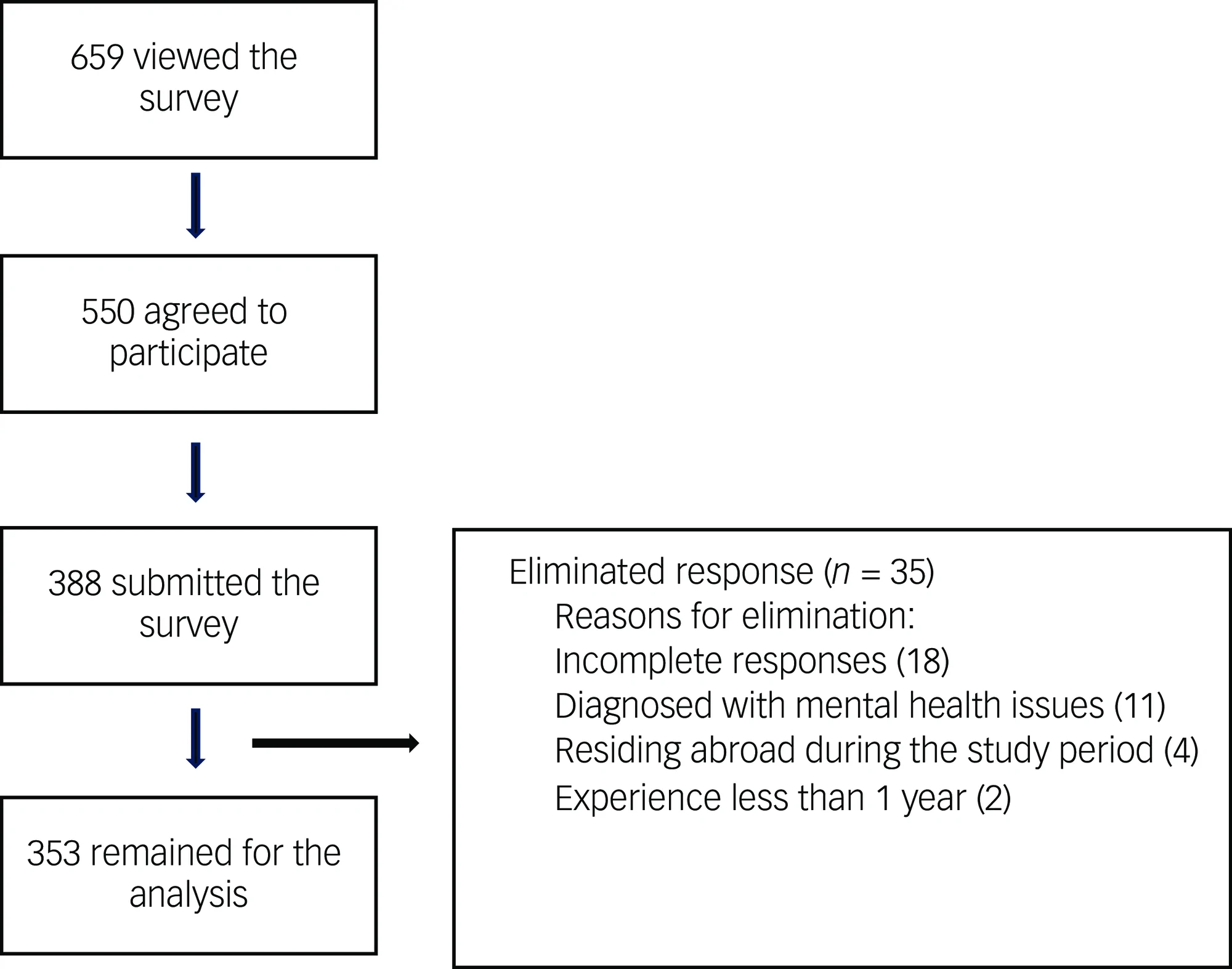
Participants flow chart.

To encourage participation and minimise bias, the questionnaire was carefully worded using neutral language. Recruitment posts were made periodically (once per week) to maximise participant diversity and reach, clearly describing the inclusion and exclusion criteria and the importance of this study. LimeSurvey has built-in settings to prevent duplicate responses. The confidentiality and anonymity of participants were emphasised in all communications. Following the exclusion of participants with incomplete responses (18), with diagnosed mental health issues (11), residing abroad during the study period (4) and having work experience of less than 1 year (2), 353 valid responses were included in the analyses (Fig. 1).

### Study variables

In this study, the independent variables were work characteristics that encompass three elements, namely job characteristics, social characteristics and organisational characteristics.^10^

The core components of job characteristics are grounded in Hackman and Oldham’s Job Characteristics Model (JCM), which includes autonomy, task identity, skill variety, task significance and feedback, which influence how an employee views their work and its significance.^11^ While the original JCM includes task identity, this was substituted with task variety to better reflect the nature of participants’ job roles in the study context, and to capture broader variability in daily work tasks. A similar substitution has been made in previous research.^10,12^ Social characteristics include social support and interdependence, such as support and relationships with supervisors and colleagues; interaction outside the organisation, such as friendly relationships and support from people in the community; and interpersonal outcomes, such as patient satisfaction.^10,12^ Organisational characteristics include organisational support such as managerial support; and physical work environment such as workplace cleanliness, noise, temperature, workload and professional political dynamics.^10^

The questionnaire consists of items that highlight those 3 elements using a 5-point Likert scale as responses, where 1 represents ‘strongly disagree’ and 5 represents ‘strongly agree’. Job characteristics, social characteristics and organisational characteristics comprise 9, 5 and 18 items, respectively. The scores for each scale were averaged to compute total scores for job characteristics, social characteristics and organisational characteristics.

A previous study among Bangladeshi physicians validated these scales with good reliability and internal consistency, where Cronbach’s alpha-values were 0.72, 0.72 and 0.77 for job characteristics, social characteristics and organisational characteristics, respectively.^10^

Mental health as the dependent variable was assessed using the Depression, Anxiety, and Stress Scale (DASS-21) developed by Lovibond and Lovibond.^20^ This scale includes 21 items divided into 3 subscales (7 items each for depression, anxiety and stress). Participants rated the severity of their experiences on a 4-point Likert scale ranging from 0 (‘Did not apply to me at all’) to 3 (‘Applied to me very much or often’). The final scores for each subscale were calculated by summing the items and multiplying by 2.^20^ Conventionally, the scores are distributed in five categories for each subscale, namely normal, mild, moderate, severe and extremely severe (see Supplementary Material 2). In this study, the outcomes were categorised into two groups based on established cut-off scores: normal and elevated states of depression (≥10), anxiety (≥8) and stress (≥15).^21^ The DASS-21 scale has previously been validated and used in Bangladesh, demonstrating strong reliability.^7,22^ Cronbach’s alpha-values for depression, anxiety and stress were 0.99, 0.96 and 0.97, respectively.^7^

Covariates include demographic and work-related factors based on prior studies, including age, gender, marital status, living status, organisation type, specialisation, physician type, private/dual practice, working area, comorbidities and work experience.^4,10,17^

### Data analysis

The data were analysed using the Statistical Package for Social Sciences (SPSS version 29 for Windows), with the significance level set at *α* < 0.05 and a 95% confidence interval. Descriptive statistics were employed to summarise the data; frequencies and percentages were calculated for categorical variables, while medians and interquartile ranges were computed for continuous variables.

To explore differences between male and female physicians, analyses were stratified by gender. The chi-square test and Fisher’s exact test were used for categorical variables. Because we found non-normally distributed data using the Kolmogorov–Smirnov test (*P* < 0.05), we further performed the Mann–Whitney *U*-test for continuous variables. Binary logistic regression analyses were then performed to examine the associations between work characteristics and mental health outcomes, controlling for potential confounders. To ensure the validity of the regression models, multicollinearity was assessed using variance inflation factors of <10.

## Results

We obtained results from a total of 353 physicians, comprising 204 (57.8%) males and 149 (42.2%) females, from all 8 divisions of Bangladesh; 59.5% were from the capital city, Dhaka.

Table 1 presents the demographic characteristics of participants stratified by gender. Of all participants, 173 (49.0%) were aged 24–29 years, 240 (68.0%) were married, 291 (82.4%) lived with family/colleague(s), 238 (67.4%) worked in the private sector, 326 (92.4%) had a Bachelor of Medicine and Bachelor of Surgery (MBBS) degree and 196 (55.8%) had private practices, with a median work experience of 4.0 years. Gender differences were statistically significant in terms of specialisation (*P* = 0.030), physician type (*P* < 0.001) and engagement in private or dual practice (*P* < 0.001). Conversely, no significant differences among male and female physicians were found in our sample regarding age group, marital status, living conditions, organisation type, working area, comorbidities or work experience.

**Table 1:** Gender-stratified demographic characteristics of participants (*N* = 353) Table 1 long description.

Variables	Overall (*N* = 353)	Male physicians (*N* = 204)	Female physicians (*N* = 149)	*P*-value
Age group, *n* (%)				
24–29 years	173 (49.0)	96 (47.1)	77 (51.7)	0.690
30–35 years	153 (43.3)	92 (45.1)	61 (40.9)	
36 years and over	27 (7.7)	16 (7.8)	11 (7.4)	
Marital status, *n* (%)				
Single	106 (30.0)	63 (30.9)	43 (28.9)	0.918^a^
Married	240 (68.0)	137 (67.1)	103 (69.1)	
Divorced/separated/widowed	7 (2.0)	4 (2.0)	3 (2.0)	
Living conditions, *n* (%)				
Alone	62 (17.6)	41 (20.1)	21 (14.1)	0.140
With family/colleague(s)	291 (82.4)	163 (79.9)	128 (85.9)	
Organisation type, *n* (%)				
Public sector	115 (32.6)	72 (35.3)	43 (28.9)	0.203
Private sector	238 (67.4)	132 (64.7)	106 (71.1)	
Specialisation, *n* (%)				
Non-specialised	252 (71.4)	151 (74)	101 (67.8)	**0.030**
General dental practitioner	22 (6.2)	7 (3.4)	15 (10.1)	
Specialised	79 (22.4)	46 (22.5)	33 (22.1)	
Physician type, *n* (%)				
MBBS doctor	326 (92.4)	197 (96.6)	129 (86.6)	**<0.001^a^**
Dentist	27 (7.6)	7 (3.4)	20 (13.4)	
Private/dual practice, *n* (%)				
Yes	196 (55.5)	132 (64.7)	64 (43.0)	**<0.001**
No	157 (44.5)	72 (35.3)	85 (57.0)	
Working area, *n* (%)				
Barisal	13 (3.7)	9 (4.4)	4 (2.7)	0.510^a^
Chattagram	40 (11.3)	29 (14.2)	11 (7.4)	
Dhaka	210 (59.5)	116 (56.9)	94 (63.1)	
Rajshahi	33 (9.3)	17 (8.3)	16 (10.7)	
Rangpur	10 (2.8)	7 (3.4)	3 (2.0)	
Mymensingh	11 (3.1)	6 (2.9)	5 (3.4)	
Sylhet	10 (2.8)	6 (2.9)	4 (2.7)	
Khulna	26 (7.4)	14 (6.9)	12 (8.1)	
Comorbidities, *n* (%)				
No	257 (72.8)	150 (73.5)	107 (71.8)	0.720
Yes	96 (27.2)	54 (26.5)	42 (28.2)	
Work experience, median (IQR)	4.0 (2.0–6.0)	4.0 (2.5–7.0)	4.0 (2.0–6.0)	0.191

MBBS, Bachelor of Medicine and Bachelor of Surgery; IQR, interquartile range.

a. Fisher’s exact test.

Significance shown in bold (*P* < 0.05), based on the Mann–Whitney *U*-test for continuous variables and either the chi-square test or Fisher’s exact test for categorical variables.

Table 2 presents data on depression, anxiety and stress across the variables. Among covariates, age, living conditions and work experience were significantly associated with all outcome variables. Furthermore, working area was associated with depression and stress whereas gender was associated with anxiety and stress. Private/dual practice was associated only with stress. Moreover, all work characteristics variables were statistically significant, with depression, anxiety and stress based on non-parametric analyses.

**Table 2:** Depression, anxiety and stress across the variables (*N* = 353) Table 2 long description.

Variables	Depression		Anxiety		Stress		*P*-value
	Elevated	Normal	Elevated	Normal	Elevated	Normal	
Age group, *n* (%)							
24–29 years	127 (73.4)	46 (26.6)	109 (63.0)	64 (37.0)	95 (54.9)	78 (45.1)	**0.010**; **0.010**; **0.020**
30–35 years	105 (68.6)	48 (31.4)	96 (62.7)	57 (37.3)	79 (51.6)	74 (48.4)	
36 years and over	12 (44.4)	15 (55.6)	9 (33.3)	18 (66.7)	7 (25.9)	20 (74.1)	
Gender, *n* (%)							
Male	133 (65.2)	71 (34.8)	110 (53.9)	94 (46.1)	94 (46.1)	110 (53.9)	0.060; **0.010**; **0.020**
Female	111 (74.5)	38 (25.5)	104 (69.8)	45 (30.2)	87 (58.4)	62 (41.6)	
Marital status, *n* (%)							
Single	82 (77.4)	24 (22.6)	71 (67.0)	35 (33.0)	59 (55.7)	47 (44.3)	0.080; 0.180; 0.270
Married	157 (65.4)	83 (34.6)	140 (58.3)	100 (41.7)	117 (48.8)	123 (51.2)	
Divorced/separated/widowed	5 (71.4)	2 (28.6)	3 (42.9)	4 (57.1)	5 (71.4)	2 (28.6)	
Living conditions, *n* (%)							
Alone	50 (80.6)	12 (19.4)	45 (72.6)	17 (27.4)	40 (64.5)	22 (35.5)	**0.030**; **0.030**; **0.020**
With family/colleague(s)	194 (66.7)	97 (33.3)	169 (58.1)	122 (41.9)	141 (48.5)	150 (51.5)	
Organisation type, *n* (%)							
Public sector	78 (67.8)	37 (32.2)	71 (61.7)	44 (38.3)	61 (53.0)	54 (47.0)	0.710; 0.770; 0.640
Private sector	166 (69.7)	72 (30.3)	143 (60.1)	95 (39.9)	120 (50.4)	118 (49.6)	
Specialisation, *n* (%)							
Non-specialised	185 (70.6)	77 (29.4)	162 (61.8)	100 (38.2)	140 (53.4)	122 (46.6)	0.380; 0.350; 0.350
General dental practitioner	16 (72.7)	6 (27.3)	15 (68.2)	7 (31.8)	9 (40.9)	13 (59.1)	
Specialised	43 (62.3)	26 (37.7)	37 (53.6)	32 (46.4)	32 (46.4)	37 (53.6)	
Physician type, *n* (%)							
Medical doctor	226 (69.3)	100 (30.7)	197 (60.4)	129 (39.6)	170 (52.1)	156 (47.9)	0.770; 0.790; 0.250^a^
Dentist	18 (66.7)	9 (33.3)	17 (63.0)	10 (37.0)	11 (40.7)	16 (59.3)	
Private/dual practice, *n* (%)							
Yes	130 (66.3)	66 (33.7)	113 (57.7)	83 (42.3)	88 (44.9)	108 (55.1)	0.200; 0.200; **0.010**
No	114 (72.6)	43 (27.4)	101 (64.3)	56 (35.7)	93 (59.2)	64 (40.8)	
Working area, *n* (%)							
Barisal	11 (84.6)	2 (15.4)	12 (92.3)	1 (7.7)	9 (69.2)	4 (30.8)	**0.010**; 0.060; **0.030** ^a^
Chattagram	24 (60.0)	16 (40.0)	23 (57.5)	17 (42.5)	21 (52.5)	19 (47.5)	
Dhaka	153 (72.9)	57 (27.1)	124 (59.0)	86 (41.0)	108 (51.4)	102 (48.6)	
Rajshahi	26 (78.8)	7 (21.2)	23 (69.7)	10 (30.3)	16 (48.5)	17 (51.5)	
Rangpur	5 (50.0)	5 (50.0)	5 (50.0)	5 (50.0)	4 (40.0)	6 (60.0)	
Mymensingh	6 (54.5)	5 (45.5)	5 (45.5)	6 (54.5)	7 (63.6)	4 (36.4)	
Sylhet	8 (80.0)	2 (20.0)	9 (90.0)	1 (10.0)	9 (90.0)	1 (10.0)	
Khulna	11 (42.3)	15 (57.7)	13 (50.0)	13 (50.0)	7 (26.9)	19 (73.1)	
Comorbidities, *n* (%)							
No	196 (70.0)	80 (29.0)	167 (60.5)	109 (39.5)	140 (50.7)	136 (49.3)	0.140; 0.930; 0.690
Yes	48 (62.3)	29 (37.7)	47 (61.0)	30 (39.0)	41 (53.2)	36 (46.8)	
Work experience, median (IQR)	4.0 (2.0–6.0)	5.0 (3.0–7.5)	4.0 (2.0–6.0)	4.5 (3.0–7.0)	4.0 (2.0–6.0)	4.3 (3.0–7.0)	**0.004**; **0.037**; **0.011**
Job characteristics, median (IQR)	3.4 (3.1–3.7)	3.7 (3.4–4.0)	3.4 (3.1–3.8)	3.7 (3.3–4.0)	3.4 (3.1–3.7)	3.7 (3.4–4.0)	**<0.001**; **<0.001**; **<0.001**
Social characteristics, median (IQR)	3.6 (3.2–4.0)	4.0 (3.7–4.4)	3.6 (3.2–4.0)	4.0 (3.6–4.2)	3.6 (3.1–4.0)	4.0 (3.6–4.2)	**<0.001**; **<0.001**; **<0.001**
Organisational characteristics, median (IQR)	3.0 (2.6–3.4)	3.3 (3.0–3.6)	3.0 (2.6–3.4)	3.2 (2.8–3.6)	2.9 (2.5–3.3)	3.3 (2.9–3.6)	**<0.001**; **<0.001**; **<0.001**

IQR, interquartile range.

a. Fisher’s exact test.

Significance shown in bold (*P* < 0.05), based on the Mann–Whitney *U*-test for continuous variables and either the chi-square test or Fisher’s exact test for categorical variables.

Table 3 presents the gender-stratified analysis of independent and dependent variables, showing how both are associated with gender and differ significantly between the genders. The median scores of job characteristics and social characteristics were significantly higher among male physicians, whereas depression, anxiety and stress were considerably higher among female physicians.

**Table 3:** Gender-stratified analysis of independent and dependent variables (*N* = 353) Table 3 long description.

Variables	Overall (*N* = 353)	Male physicians (*n* = 204)	Female physicians (*n* = 149)	*P*-value
**Work characteristics**, median (IQR)				
Job characteristics	3.6 (3.2–3.9)	3.6 (3.3–3.9)	3.4 (3.1–3.9)	**0.016**
Social characteristics	3.8 (3.4–4.0)	3.8 (3.4–4.2)	3.6 (3.2–4.0)	**0.012**
Organisational characteristics	3.1 (2.7–3.5)	3.1 (2.7–3.5)	3.1 (2.7–3.4)	0.530
**Mental health outcomes**, *n* (%)				
Depression	244 (69.1)	133 (65.2)	111 (74.5)	0.062
Anxiety	214 (60.6)	110 (53.9)	104 (69.8)	**0.003**
Stress	181 (51.3)	94 (46.1)	87 (58.4)	**0.022**

IQR, interquartile range.

Significance shown in bold (*P* < 0.05), based on the Mann–Whitney *U*-test for continuous variables and either the chi-square test or Fisher’s exact test for categorical variables.

Table 4 presents the results of the binary logistic regression analysis for all participants (*N* = 353); these data fit perfectly with this model. We found that social characteristics were significantly and inversely associated with depression (odds ratio 0.37 (0.20–0.71)), anxiety (odds ratio 0.53 (0.30–0.92)) and stress (odds ratio 0.45 (0.26–0.81)). In addition, organisational characteristics were significantly and inversely associated with stress (odds ratio 0.42 (0.24–0.74)), but not with anxiety or depression. In this paradigm, job characteristics were not substantially associated with stress, anxiety or depression.

**Table 4:** Binary logistic regression analysis for all participants (*N* = 353) Table 4 long description.

Independent variable	Depression Odds ratio (95% CI)	Anxiety Odds ratio (95% CI)	Stress Odds ratio (95% CI)
Work characteristics			
Job characteristics	0.67 (0.33–1.35)	0.83 (0.45–1.56)	0.77 (0.40–1.48)
Social characteristics	0.37 (0.20–0.71)**	0.53 (0.30–0.92)*	0.45 (0.26–0.81)**
Organisational characteristics	0.59 (0.32–1.06)	0.73 (0.43–1.25);	0.42 (0.24–0.74)**
Age group			
24–29 years	Ref.	Ref.	Ref.
30–35 years	1.22 (0.63–2.34)	1.41 (0.78–2.54)	1.20 (0.66–2.19)
36 years and over	0.96 (0.20–4.68)	0.63 (0.14–2.84)	0.58 (0.12–2.86)
Gender			
Male	Ref.	Ref.	Ref.
Female	1.34 (0.78–2.31)	2.00 (1.22–3.29)**	1.52 (0.92–2.51)
Living conditions			
Alone	Ref.	Ref.	Ref.
With family/colleague(s)	0.56 (0.26–1.21)	0.60 (0.31–1.16)	0.66 (0.35–1.26)
Private/dual practice			
Yes	Ref.	Ref.	Ref.
No	0.86 (0.50–1.49)	0.91 (0.55–1.49)	1.34 (0.81–2.22)
Work experience	0.90 (0.80–1.01)	0.95 (0.85–1.05)	0.94 (0.84–1.05)
Working area	0.14 (0.02–0.96)*	0.07 (0.01–0.76)*	0.21 (0.04–1.06)

Ref., reference.

Significant results are in bold.

*P < 0.05, **P < 0.01.

Table 5 presents the results of the gender-stratified binary logistic regression analysis. According to the results, the associations differ between male and female physicians; these data fit perfectly with this model. We adjusted for age, living conditions, private practice and work experience, because these covariates were significantly associated with outcome variables in the non-parametric tests. In this gender-stratified model, the covariate working area was excluded to avoid statistical issues arising from small call counts for either gender in some areas. For male physicians, social characteristics were a significant protective factor for stress (adjusted odds ratio 0.43 (0.19–0.97)). Organisational characteristics were also protective, showing significant inverse associations with depression (adjusted odds ratio 0.42 (0.19–0.90)), anxiety (adjusted odds ratio 0.44 (0.21–0.91)) and stress (adjusted odds ratio 0.42 (0.20–0.89)).

**Table 5:** Gender-stratified binary logistic regression analysis (*N* = 353) Table 5 long description.

Independent variable	Depression Adjusted odds ratio (95% CI)	Anxiety Adjusted odds ratio (95% CI)	Stress Adjusted odds ratio (95% CI)
Male physicians			
Job characteristics	0.73 (0.31–1.75)	0.74 (0.32–1.68)	0.56 (0.24–1.32)
Social characteristics	0.51 (0.21–1.17)	0.53 (0.24–1.17)	0.43 (0.19–0.97)*
Organisational characteristics	0.42 (0.19–0.90)*	0.44 (0.21–0.91)*	0.42 (0.20–0.89)*
Female physicians			
Job characteristics	0.71 (0.22–2.32)	0.87 (0.32–2.35)	1.02 (0.39–2.66)
Social characteristics	0.30 (0.12–0.78)*	0.54 (0.25–1.18)	0.55 (0.26–1.18)
Organisational characteristics	1.12 (0.42–2.94)	1.56 (0.66–3.67)	0.47 (0.20–1.10)

Adjusted odds ratio, adjusted for age, living conditions, private practice and work experience.

Significant results are in bold.

*P < 0.05.

For female physicians the findings were different. Only social characteristics demonstrated a significant inverse association with depression (adjusted odds ratio 0.30 (0.12–0.78)), while no significant associations were found for job characteristics or organisational characteristics.

## Discussion

This study investigated the relationships between different work characteristics, namely job characteristics, social characteristics and organisational characteristics, and mental health outcomes (depression, anxiety and stress) among physicians. It also examined potential differences in the dimensions of work characteristics among male and female physicians. The following discussion interprets our findings in the context of the literature, explores potential explanations and makes recommendations for future directions for both research and practice.

Our findings indicate that, based on the DASS-21 cut-off points, the prevalence rates of depression, anxiety and stress among Bangladeshi physicians were 69.1, 60.6 and 51.3%, respectively. This indicates that 69.1% of physicians experienced depression, 60.6% experienced anxiety and 51.3% experienced stress at varying levels of severity, from mild to extremely severe. In line with our findings, a previous study that used the DASS-21 scale reported the prevalence of depression among physicians as 55.3%, anxiety as 35.2% and stress as 48.4%.^7^ However, this variation can be attributed to differences in cut-off criteria, because that study reported prevalence based only on severe and extremely severe cases of depression, anxiety and stress while categorising normal, mild and moderate cases as normal.^7^ Prevalence increased substantially not only in Bangladesh but also worldwide. A systematic review reporting the global prevalence of depression and anxiety disorders in 204 countries showed a substantial increase in the prevalence of MDD and anxiety disorders.^2^

In the present study, a significant gender disparity was observed regarding the prevalence of anxiety and stress. Female physicians reported a higher prevalence of depression (74.5 *v.* 65.2%), anxiety (69.8 *v.* 53.9%) and stress (58.4 *v.* 46.1%) than male physicians. These findings are consistent with previous studies in similar contexts showing that women experience a greater burden of both MDD and anxiety disorders, not only in LMICs but also globally, regardless of geographical location, race or ethnicity.^2,4,7,23,24^ This phenomenon is often attributed to a combination of factors, including biological influences like sex hormones, as well as psychological stressors such as higher rates of interpersonal stressors, lack of gender equality and discrimination, etc.^24^

Regarding work characteristics, we found that social characteristics (e.g. support and relationships with supervisors and colleagues) were negatively associated with depression, anxiety and stress. This suggests that better social support in the workplace and good relationships with both peers and patients are beneficial for psychological well-being. In addition, organisational characteristics were negatively associated only with stress, indicating that good organisational factors (e.g. organisational support, healthy physical work environment) were helping in mitigating stress. However, job characteristics did not show any significant associations with depression, anxiety or stress, which may raise scepticism about presumptions regarding its protective function. This finding implies that the influence of job characteristics on physician mental health may not be as important as that of social or organisational characteristics.

The negative association between social characteristics and mental health outcomes in this study aligns with previous studies recognising social support as an essential safeguard against emotional distress, burnout and depression and promoting overall well-being, particularly in stressful situations.^25,26^ Similarly, Fu et al highlighted the importance of social support, especially from supervisors, in helping physicians build confidence and a positive self-image, which can protect against depression.^27^ Support from peers is also vital in mitigating mental health issues among healthcare workers. While supervisors are often responsible for providing mentorship and instrumental support, co-workers are more accessible for emotional support, fostering a positive work atmosphere and alleviating isolation and stress. The well-being of physicians can be further improved by interpersonal connections and positive team dynamics, underscoring the critical role of peer support in promoting mental health.^28,29^

However, our study also revealed that the association of social characteristics and mental health varies by gender. For male physicians it was associated with stress, whereas for female physicians it was linked to depression. In this context, Smith et al reported that mental health in men is often characterised by externalising symptoms, which may be revealed as stress in professional settings, whereas internalising disorders (e.g. depression) are more common among women.^30^ Additionally, owing to societal expectations, male physicians might feel career-related pressure, which can be mitigated by higher social characteristics. On the other hand, female physicians tend to experience a different set of challenges related to social characteristics, which often leads to increased depressive symptoms.^7,31^ Despite their professional success, female physicians still face patriarchal expectations of fulfilling domestic roles, such as home management and child-rearing, leading to added pressure from balancing careers and families.^18,32^ Social support from partners or family has a significant role in protecting women against burnout. On the other hand, social support from colleagues and involvement in workplace decision-making are more important factors for men.^33^ Although the role of social characteristics differs by gender, their enhancement can be one of the main target areas for protecting and promoting mental health among physicians.^34^

Furthermore, organisational characteristics (e.g. organisational support) are particularly prominent in a high-stress occupation such as healthcare. One study during the COVID-19 pandemic revealed that the mental health of healthcare workers was strongly influenced by their perceived organisational support, underscoring the necessity for organisations to provide sufficient support networks.^35^ Another study, in the Bangladeshi healthcare context, revealed that organisational characteristics exert greater influence as a stressor for burnout among physicians in the public sector than among those in the private sector, whereas social characteristics play a more significant role in mitigating burnout in the private sector.^10^ Notably, in our study, organisational characteristics play a substantial role in influencing the mental health of male physicians, because these were associated with depression, anxiety and stress. In contrast, no association was observed for female physicians. Male physicians may place greater importance on workplace factors, viewing them as key to their identity and success and making those factors more impactful for their overall mental health. On the other hand, by navigating through a male-dominated society and workplaces, female physicians may have developed coping strategies for the organisational impact on their mental health.

Nevertheless, no significance was found between job characteristics and mental health in our study, although the protective role of job characteristics (e.g. workplace autonomy, task variety, etc.) has been well documented in previous research.^14,36^ Workplace autonomy, which is a core component of the JD-R model, significantly reduces stress, increases job satisfaction and alleviates anxiety and depressive symptoms, whereas task variety can reduce monotonous work environments and boredom.^37^ In contrast, Steyn and Vawda argue that job characteristics are more likely to influence job satisfaction than overall mental health.^38^

This study incorporated a broad range of work-related predictors to assess the relationship between work characteristics and mental health among physicians, highlighting the critical role of social support and relationships in influencing mental health in this profession. The inclusion of a diverse set of work-related factors has revealed important insights and enhanced our understanding of how those factors are associated with mental health, as well as different gender experiences in the LMIC context. The online nature of this survey facilitated rapid and efficient data collection with a wide reach, and its anonymous nature allowed the respondents to feel confident in their ability to answer honestly, which may have helped to reduce social desirability bias. However, it is important to acknowledge certain limitations of this study. First, the cross-sectional design restricted its ability to establish causal relationships. Second, for self-reported measures, we obtained only subjective perceptions rather than objective measures for cross-validation, which makes the objectivity of our results somewhat limited. Third, the results of this study may have limited generalisability because the web-based survey might have introduced sample bias by leaving out those who did not have internet access or were not active on social media. Additionally, the focus on the total scores of the independent variables may have masked the contribution of specific subdomains. Despite these limitations, this study provides important findings that should be taken into consideration when tailoring preventive or therapeutic mental health interventions in the healthcare context.

Work characteristics in the Bangladeshi health system require significant improvement to ensure mental well-being among physicians. Male and female physicians perceive differences in their workplaces. While social characteristics are strongly linked to elevated levels of depression, anxiety and stress overall, these are related only to stress in males and to depression in females. In addition, organisational characteristics are strongly associated with mental health outcomes in male physicians but not in their female counterparts. Targeted interventions to improve social support and organisational policies could reduce the mental health burden among physicians. Finally, future research should further explore the underlying causes and mechanisms behind these gendered experiences and perceptions, to develop comprehensive intervention strategies for enhancement of physician well-being and reduction in the mental health burden in the healthcare sector.

## Supporting information

Alpona et al. supplementary material 1 Alpona et al. supplementary material

Alpona et al. supplementary material 2 Alpona et al. supplementary material

## Data Availability

The data contain sensitive information. However, the corresponding author, T.A., will make these available upon reasonable request.

## Supplementary material

The supplementary material is available online at https://doi.org/10.1192/bji.2025.10075.

## Table 1 Long description

A table comparing demographic characteristics of male and female physicians. The table has 12 rows and 6 columns. The columns are labeled Variables, Overall (N = 353), Male physicians (N = 204), Female physicians (N = 149), and P-value. The rows are labeled with different variables such as Age group, Marital status, Living conditions, Organisation type, Specialisation, Physician type, Private/dual practice, Working area, and Comorbidities. Each row provides percentages for each variable across the different groups. Notable trends include significant gender differences in specialisation, physician type, and engagement in private or dual practice.

Navigate back to Table 1.

## Table 2 Long description

A table comparing depression, anxiety, and stress across different variables. The table has 18 rows and 10 columns. Column headers include Age group, Gender, Marital status, Living conditions, Organisation type, Specialisation, Physician type, Private/dual practice, Working area, and Comorbidities. Row labels include categories like 24-29 years, Male, Single, Alone, Public sector, Non-specialised, Medical doctor, Yes, Barisal, and No. The table presents percentages of elevated and normal levels of depression, anxiety, and stress for each category, along with P-values indicating statistical significance. Notable trends include significant associations of age, living conditions, and work experience with all outcome variables, and specific associations of working area with depression and stress, gender with anxiety and stress, and private/dual practice with stress. All work characteristics variables were statistically significant with depression, anxiety, and stress based on non-parametric analyses.

Navigate back to Table 2.

## Table 3 Long description

The table presents a gender-stratified analysis of independent and dependent variables for 353 physicians, divided into male (204) and female (149) groups. It has 2 sections: Work characteristics and Mental health outcomes. Work characteristics include Job characteristics, Social characteristics, and Organisational characteristics, each with median scores and interquartile ranges (IQR). Mental health outcomes include percentages of physicians experiencing Depression, Anxiety, and Stress. Notable trends include higher median scores for job and social characteristics among male physicians and higher percentages of depression, anxiety, and stress among female physicians. P-values indicate the significance of differences between genders for each variable.

Navigate back to Table 3.

## Table 4 Long description

The table presents the results of a binary logistic regression analysis for all participants, focusing on the odds ratios of various independent variables on depression, anxiety, and stress. It includes 10 rows and 4 columns. The columns are labeled 'Independent variable', 'Depression Odds ratio (95% CI)', 'Anxiety Odds ratio (95% CI)', and 'Stress Odds ratio (95% CI)'. The rows are labeled with different independent variables such as work characteristics, age group, gender, living conditions, private/dual practice, work experience, and working area. Each cell contains the odds ratio and confidence interval for the respective variable and condition. Notable trends include significant inverse associations between social characteristics and depression, anxiety, and stress, as well as between organisational characteristics and stress.

Navigate back to Table 4.

## Table 5 Long description

A table comparing depression, anxiety, and stress factors among male and female physicians. The table has 3 columns and 8 rows. The columns are labeled Independent variable, Depression Adjusted odds ratio (95% CI), Anxiety Adjusted odds ratio (95% CI), and Stress Adjusted odds ratio (95% CI). The rows are labeled Male physicians and Female physicians, with sub-rows for Job characteristics, Social characteristics, and Organisational characteristics. For male physicians, the values are: Job characteristics: Depression 0.73 (0.31–1.75), Anxiety 0.74 (0.32–1.68), Stress 0.56 (0.24–1.32); Social characteristics: Depression 0.51 (0.21–1.17), Anxiety 0.53 (0.24–1.17), Stress 0.43 (0.19–0.97); Organisational characteristics: Depression 0.42 (0.19–0.90), Anxiety 0.44 (0.21–0.91), Stress 0.42 (0.20–0.89). For female physicians, the values are: Job characteristics: Depression 0.71 (0.22–2.32), Anxiety 0.87 (0.32–2.35), Stress 1.02 (0.39–2.66); Social characteristics: Depression 0.30 (0.12–0.78), Anxiety 0.54 (0.25–1.18), Stress 0.55 (0.26–1.18); Organisational characteristics: Depression 1.12 (0.42–2.94), Anxiety 1.56 (0.66–3.67), Stress 0.47 (0.20–1.10).

Navigate back to Table 5.
