# Supplementary material for: Work characteristics and mental health among physicians in Bangladesh: highlighting gendered perspectives
Source: BJPsych Int. 2025 Dec 22;23(4):189–99. doi: 10.1192/bji.2025.10075 (PMC13732221; doi:10.1192/bji.2025.10075)
Supplement: Supplementary file 1 [file S2056474025100755sup001.docx]

**Supplemental material 1**

**Study questionnaire**

**Section 1: Personal and professional background**

| **No.** | **Question** | **Response** | **Code** |
| --- | --- | --- | --- |
| 1.1 | Your email address |  | |
| 1.2 | What is your age? | …… Years | |
| 1.3 | Gender | Male | 1 |
|  |  | Female | 2 |
| 1.4 | Marital status | Single | 1 |
|  |  | Married | 2 |
|  |  | Other (Comment) | 3 |
| 1.5 | Living condition | Living alone | 1 |
|  |  | Living with family | 2 |
|  |  | Other (Comment) | 3 |
| 1.6 | Are you currently working as a doctor in Bangladesh? | Yes | 1 |
|  |  | No | 2 |
| 1.7 | Your affiliation (Eg. XYZ hospital/clinic) | Hospital name: | # |
| 1.8 | Title of your current position | Medical Officer | 1 |
|  |  | Junior Consultant | 2 |
|  |  | Senior Consultant | 3 |
|  |  | Residential Medical Officer | 4 |
|  |  | Lecturer | 5 |
|  |  | Assistant Surgeon | 6 |
|  |  | Assistant Dental surgeon | 7 |
|  |  | Other (Comment) | # |
| 1.9 | What is your specialization? (Please write down only BMDC-recognized specializations) | General Physician | 1 |
|  |  | BDS | 2 |
|  |  | Specialized in……(Comment) | 3 |
| 1.10 | In which sector you are working: | Public sector | 1 |
|  |  | Private sector | 2 |
| 1.11 | How many years have you worked as a doctor? | ………….Years | |
| 1.12 | If you are a public doctor: Where are you posted now? | Sub-district Health Complex | 1 |
|  |  | District hospital | 2 |
|  |  | Medical college hospital | 3 |
|  |  | Not Public (Comment) |  |
|  |  | Others (eg. PGourse) (Comment) | 4 |
| 1.13 | Are you doing private practice or working part-time in other private clinics or hospitals? | Yes | 1 |
|  |  | No | 2 |
| 1.14 | Please select your working division. | Dhaka / Chittagong / Khulna / Rajshahi / Sylhet/ Mymensingh/ Barisal / Rangpur | # |
| 1.15 | Please select your working district | Name list districts (64) | # |

**Section 2: Work Characteristics domains**

***All statements/ questions are measured on a 5-point rating scale: 1 = strongly disagree, 2 = disagree, 3 = neither agree nor disagree, 4 = agree and 5 = strongly agree. Kindly choose one of the five options that you consider to be accurate.***

**2.1 Job characteristics**

| 2.1.1 | I have adequate freedom to make my duty schedule | 1 | 2 | 3 | 4 | 5 |
| --- | --- | --- | --- | --- | --- | --- |
| 2.1.2 | I have the freedom of choosing methods to treat patients *(such as advising diagnostic tests, medicines, and surgical/ conservative treatment)* | 1 | 2 | 3 | 4 | 5 |
| 2.1.3 | I do the same tasks repeatedly in everyday practice *(e.g. only outdoor or indoor or emergency duties or operations etc.)* | 1 | 2 | 3 | 4 | 5 |
| 2.1.4 | I have the opportunity to do different types of tasks every day (*e.g. indoor, outdoor, emergency, surgery, patient discharge, etc.)* | 1 | 2 | 3 | 4 | 5 |
| 2.1.5 | I enjoy doing the same tasks repeatedly | 1 | 2 | 3 | 4 | 5 |
| 2.1.6 | I enjoy different tasks in a day | 1 | 2 | 3 | 4 | 5 |
| 2.1.7 | I feel my job has a significant effect on people’s lives. | 1 | 2 | 3 | 4 | 5 |
| 2.1.8 | I often get patients’ feedback that conveys recognition of how well I am doing my job. | 1 | 2 | 3 | 4 | 5 |
| 2.1.9 | I need different types of skills to do my tasks | 1 | 2 | 3 | 4 | 5 |

**2.2 Social Characteristics**

| 2.2.1 | I get adequate support from my supervisor | 1 | 2 | 3 | 4 | 5 |
| --- | --- | --- | --- | --- | --- | --- |
| 2.2.2 | My colleagues are adequately supportive and friendly | 1 | 2 | 3 | 4 | 5 |
| 2.2.3 | My patients are adequately satisfied. | 1 | 2 | 3 | 4 | 5 |
| 2.2.4 | I have a good relationship with patients. | 1 | 2 | 3 | 4 | 5 |
| 2.2.5 | Most often I experience friendly relationships with and support from people in the community. | 1 | 2 | 3 | 4 | 5 |

**2.3 Organizational characteristics**

| **Organizational supports** | | | | | | |
| --- | --- | --- | --- | --- | --- | --- |
| 2.3.1 | I get adequate managerial support *(e.g. support from UHFPO / Civil surgeon/superintendent /director or manager of private clinic/hospital)* | 1 | 2 | 3 | 4 | 5 |
| 2.3.2 | There is a transparent system of performance evaluation in my organization. | 1 | 2 | 3 | 4 | 5 |
| 2.3.3 | There is a reward system, such as promotion and increment, which is based on performance evaluation | 1 | 2 | 3 | 4 | 5 |
| 2.3.4 | Salary and benefits are adequate in my organization | 1 | 2 | 3 | 4 | 5 |
| 2.3.5 | There is job security in my organization | 1 | 2 | 3 | 4 | 5 |
| 2.3.6 | There is an opportunity for career development | 1 | 2 | 3 | 4 | 5 |
| 2.3.7 | There is an established performance feedback system from the management level | 1 | 2 | 3 | 4 | 5 |
| 2.3.8 | There are adequate supplies of resources (e.g. equipment, drugs, and diagnostic facilities) | 1 | 2 | 3 | 4 | 5 |
| **Physical working conditions** | | | | | | |
| 2.3.9 | The building and cleanliness are adequate to do my daily tasks | 1 | 2 | 3 | 4 | 5 |
| 2.3.10 | The workplace is free from excessive noise and crowds | 1 | 2 | 3 | 4 | 5 |
| 2.3.11 | The workplace is comfortable in terms of temperature and humidity | 1 | 2 | 3 | 4 | 5 |
| 2.3.12 | Home to work-place transport is good | 1 | 2 | 3 | 4 | 5 |
| 2.3.13 | There are adequate living facilities surrounding my workplace | 1 | 2 | 3 | 4 | 5 |
| **Workload** | | | | | | |
| 2.3.14 | The quantity of tasks to complete my daily responsibilities is quite high | 1 | 2 | 3 | 4 | 5 |
| 2.3.15 | To complete daily tasks, I need to work hard under time pressure | 1 | 2 | 3 | 4 | 5 |
| **Health-professional politics** | | | | | | |
| 2.3.19 | Professional political identity is often more important for reward and career development than performance | 1 | 2 | 3 | 4 | 5 |
| 2.3.20 | Professional politics often discourage my commitment to the organization and job | 1 | 2 | 3 | 4 | 5 |
| 2.3.21 | Professional politics often hamper working relationships among colleagues | 1 | 2 | 3 | 4 | 5 |

**Section 3: Outcomes (Mental health (DASS-21))**

*Please read each statement and choose a number 0, 1, 2, or 3 which indicates how much the statement applied to you* ***over the past week****. There are no right or wrong answers. Do not spend too much time on any statement. The rating scale is as follows:*

0 = Did not apply to me at all - **NEVER**

1= Applied to me to some degree, or some of the time - **SOMETIMES**

2= Applied to me to a considerable degree or a good part of the time - **OFTEN**

3= Applied to me very much or most of the time – **ALMOST ALWAYS**

| **Ques.** | **Statement** | **Respondent’s rating** | | | |
| --- | --- | --- | --- | --- | --- |
| 3.1 | I found it hard to wind | **0** | **1** | **2** | **3** |
| 3.2 | I was aware of dryness in my mouth | **0** | **1** | **2** | **3** |
| 3.3 | I couldn’t seem to experience any positive feelings at all | **0** | **1** | **2** | **3** |
| 3.4 | I experienced breathing difficulty *(eg, excessively rapid breathing, breathlessness in the absence of physical exertion)* | **0** | **1** | **2** | **3** |
| 3.5 | I found it difficult to work up the initiative to do things | **0** | **1** | **2** | **3** |
| 3.6 | I tended to over-react to situations | **0** | **1** | **2** | **3** |
| 3.7 | I experienced trembling (eg, in my hands) | **0** | **1** | **2** | **3** |
| 3.8 | I felt that I was using a lot of nervous energy | **0** | **1** | **2** | **3** |
| 3.9 | I was worried about situations in which I might panic and make a fool of myself | **0** | **1** | **2** | **3** |
| 3.10 | I felt that I had nothing to look forward to | **0** | **1** | **2** | **3** |
| 3.11 | I found myself getting agitated | **0** | **1** | **2** | **3** |
| 3.12 | I found it difficult to relax | **0** | **1** | **2** | **3** |
| 3.13 | I felt downhearted and blue | **0** | **1** | **2** | **3** |
| 3.14 | I was intolerant of anything that kept me from getting on with what I was doing | **0** | **1** | **2** | **3** |
| 3.15 | I felt I was close to panic | **0** | **1** | **2** | **3** |
| 3.16 | I was unable to become enthusiastic about anything | **0** | **1** | **2** | **3** |
| 3.17 | I felt I wasn’t worth much as a person | **0** | **1** | **2** | **3** |
| 3.18 | I felt that I was rather touchy | **0** | **1** | **2** | **3** |
| 3.19 | I was aware of the action of my heart in the absence of physical exertion (*eg, sense of heart rate increase, heart missing a beat)* | **0** | **1** | **2** | **3** |
| 3.20 | I felt scared without any good reason | **0** | **1** | **2** | **3** |
| 3.21 | I felt that life was meaningless | **0** | **1** | **2** | **3** |

**The End**
