# Supplementary material for: Work characteristics and mental health among physicians in Bangladesh: highlighting gendered perspectives
Source: BJPsych Int. 2025 Dec 22;23(4):189–99. doi: 10.1192/bji.2025.10075 (PMC13732221; doi:10.1192/bji.2025.10075)
Supplement: Supplementary file 2 [file S2056474025100755sup002.docx]

**DASS-21 Scoring Instructions**

The DASS-21 should not be used to replace a face to face clinical interview. If you are experiencing significant emotional difficulties you should contact your GP for a referral to a qualified professional.

**Depression, Anxiety and Stress Scale - 21 Items (DASS-21)**

The Depression, Anxiety and Stress Scale - 21 Items (DASS-21) is a set of three self-report scales designed to measure the emotional states of depression, anxiety and stress.

Each of the three DASS-21 scales contains 7 items, divided into subscales with similar content. The depression scale assesses dysphoria, hopelessness, devaluation of life, self-deprecation, lack of interest / involvement, anhedonia and inertia. The anxiety scale assesses autonomic arousal, skeletal muscle effects, situational anxiety, and subjective experience of anxious affect. The stress scale is sensitive to levels of chronic non- specific arousal. It assesses difficulty relaxing, nervous arousal, and being easily upset / agitated, irritable / over-reactive and impatient. Score

The DASS-21 is based on a dimensional rather than a categorical conception of psychological disorder. The assumption on which the DASS-21 development was based (and which was confirmed by the research data) is that the differences between the depression, anxiety and the stress experienced by normal subjects and clinical populations are essentially differences of degree. The DASS-21 therefore has no direct implications for the allocation of patients to discrete diagnostic categories postulated in classificatory systems such as the DSM and ICD.

Recommended cut-off scores for conventional severity labels (normal, moderate, severe) are as follows: NB Scores on the DASS-21 will need to be multiplied by 2 to calculate the final score.

|  | **Depression** | **Anxiety** | **Stress** |
| --- | --- | --- | --- |
| Normal | 0-9 | 0-7 | 0-14 |
| Mild | 10-13 | 8-9 | 15-18 |
| Moderate | 14-20 | 10-14 | 19-25 |
| Severe | 21-27 | 15-19 | 26-33 |
| Extremely Severe | 28+ | 20+ | 34+ |

Reference: Lovibond, S.H. & Lovibond, P.F. (1995). Manual for the Depression Anxiety & Stress Scales. (2^nd^ Ed.)Sydney: Psychology Foundation.
